# Incidence and predictive factors of urinary catheter reinsertion in cardiac surgery patients: A cross-sectional study in Nanning, China

**DOI:** 10.1097/MD.0000000000042691

**Published:** 2025-06-06

**Authors:** Si-Si Deng, Sheng-Sheng Zhong, Ling Huang, Wang-Qiu Wu, Jiao Luo, Xin-Huan Lu

**Affiliations:** a Cardiac Surgery Ward, The First Affiliated Hospital of Guangxi Medical University, Nanning, Guangxi, China; b Cardiothoracic Vascular Surgery, The Guangxi International Zhuang Medicine Hospital, Nanning, Guangxi, China.

**Keywords:** cardiac surgery, incidence, nursing, risk factor, urinary catheter reinsertion

## Abstract

This study investigates the incidence and risk factors associated with the reinsertion of urinary catheters in post-cardiac surgery patients. Conducted at the Cardiac Surgery Ward of a university-affiliated hospital in Nanning, China, from July to December 2021, this cross-sectional study included patients aged 18 years and older undergoing cardiac surgery. Excluded were individuals with genitourinary issues, prostate surgery history, urostomy, cognitive impairment, mental illness, chronic indwelling catheter requirements, hemodialysis, or undergoing interventional surgery. Data on demographic and clinical characteristics were collected to perform multivariable logistic regression to identify risk factors for urinary catheter reinsertion. In our study of 254 cardiac surgery patients, 21 (8.26%) required urinary catheter reinsertion. Notable differences were not seen in age, gender, or body mass index between the reinsertion and no reinsertion groups. However, diabetes was significantly more prevalent in the reinsertion group (19%) compared to the no reinsertion group (3%) (*P* = .001), with diabetic patients also experiencing longer operative times and older age. The median urinary catheter duration was significantly shorter in the reinsertion group (35.03 hours) versus 60.65 hours for those without reinsertion (*P* = .034). Early catheter removal within the first postoperative day notably increased reinsertion rates, with 52.4% of early removals requiring reinsertion compared to 23.2% of later removals (*P* = .003). Multivariable logistic regression highlighted key risk factors: each additional year of age increased reinsertion risk by 3.6% (OR = 1.036, *P* = .023), and diabetic patients were approximately 8.8 times more likely to require reinsertion (OR = 8.755, *P* = .004). Early catheter removal was associated with an 8.6-fold increase in reinsertion likelihood (OR = 8.570, *P* = .001). Our findings emphasize the need for personalized management strategies to prevent urinary catheter reinsertion in cardiac surgery patients, particularly among older individuals, those with diabetes, or whose catheters are removed early post-surgery. These insights are crucial for enhancing postoperative care and minimizing complications associated with urinary catheterization.

## 1. Introduction

Urinary retention is a common postoperative complication, especially after the removal of a urinary catheter, leading to re-catheterization in certain cases. This issue is prevalent in surgical patients, with studies reporting re-catheterization rates ranging from 3.8% to 14%.^[1]^ Re-catheterization not only extends hospital stays but also increases the risk of urinary tract infection,^[2]^ contributing to patient discomfort, heightened healthcare costs, and anxiety.^[3]^

While substantial research has been conducted on re-catheterization in colorectal,^[4]^ hip,^[5]^ and thoracic surgeries,^[6]^ cardiac surgery, with its unique set of challenges, has been less examined. The discrepancy in re-catheterization rates across surgeries may be due to differing patient demographics and operative conditions.^[1]^ Postoperative urinary retention (POUR) is particularly prevalent among patients undergoing general anesthesia,^[7]^ with a notably higher incidence compared to those with regional anesthesia or epidural analgesia. Longer operative times, a common feature in cardiac surgeries, have been correlated with increased POUR incidences.^[2,8]^

A key area of ongoing debate is the optimal timing for urinary catheter removal in cardiac surgery patients. While prolonged catheterization is associated with higher urinary tract infection risks,^[9]^ its early removal, though it reduces infection risk and promotes early mobilization, can lead to urinary retention and necessitate re-catheterization.^[4]^ Thus, determining the appropriate timing for catheter removal is crucial in cardiac surgical care.

Recognizing these complexities, our study aims to investigate the incidence of urinary catheter reinsertion in adult cardiac surgical patients and identify associated risk factors. This exploration is crucial for enhancing postoperative care and managing urinary catheterization effectively in this patient group, thereby contributing significant insights into improving patient outcomes and healthcare practices in cardiac surgery.

## 2. Methods

### 2.1. Study design

This cross-sectional study, aimed at understanding urinary catheter reinsertion in cardiac surgery patients, was conducted at the Cardiac Surgery Ward of a university-affiliated hospital in Nanning, China, from July to December 2021. To determine the appropriate sample size, we employed the formula $n=\frac{u_{\alpha /2}^{2}\pi (1-\pi )}{\delta^{2}}$,^[10]^ where α is the significance level (set at 0.05), π is the estimated proportion of the attribute present in the population (assumed to be 0.5), and δ is the margin of error (set at 0.1). This calculation yielded a sample size of approximately 96, ensuring statistical robustness.

### 2.2. Study participants

During the study period, we enrolled a convenience sample of 254 patients scheduled for elective cardiac surgery. The inclusion criteria were: patients aged 18 years or older, able to communicate, and undergoing their first cardiac surgery. Exclusion criteria included patients with genitourinary issues, a history of prostate surgery or urostomy, cognitive impairment or mental illness, and those needing chronic catheterization or hemodialysis. Patients scheduled for interventional surgeries were also excluded.

### 2.3. Outcome variables

We gathered demographic and clinical data from electronic medical records, including age, gender, height, weight, medical history, diagnosis, and surgical approach. Renal function was assessed through preoperative and post-catheter removal creatinine and creatinine clearance rate (CCr). We also documented ventilator support duration, cardiopulmonary bypass and operative times, intensive care unit (ICU) stay length, total hospital days, and urinary catheter removal timing. Postoperative drainage, including chest, pericardial, and mediastinal tubes, was recorded.

Patient activities of daily living and pain intensity were assessed upon ward transfer. The Barthel index, covering aspects like defecation and urinary control, was used for daily living activities evaluation.^[11,12]^ Based on the level of independence, the charge nurses utilized the Barthel index to assign a score that reflected the patient’s ability. Pain intensity was measured using the numeric rating scale, ranging from 0 (no pain) to 10 (worst possible pain).

The primary study outcome was the incidence of urinary catheter reinsertion, with secondary outcomes focusing on risk factors for catheter reinsertion in cardiac surgery patients.

Catheter removal decisions were made by attending physicians, considering patient conditions. Re-catheterization criteria included inability to void and bladder scans showing over 500 mL of urine retention within 8 hours. We recorded the time of the second urinary catheter removal for patients who required urinary re-catheterization.

### 2.4. Data analysis

We presented descriptive data as medians and interquartile ranges. The Mann–Whitney *U* test was used for comparing non-normally distributed groups, while the Chi-square or Fisher exact test assessed categorical data differences. Logistic regression analysis identified risk factors for urinary catheter reinsertion.

### 2.5. Ethical considerations

The institutional review board of the First Affiliated Hospital of Guangxi Medical University approved this study (2023-E317-01). Informed consent was obtained from all participants, adhering to the World Medical Association Declaration of Helsinki. The STROBE (Strengthening the Reporting of Observational Studies in Epidemiology) checklist guided the study’s methodological rigor.

## 3. Results

Table 1 segregates the patients into 2 groups: those who did not require reinsertion of a cardiac device (n = 233) and those who did (n = 21). The median age of all patients studied is approximately 48.78 years, with an interquartile range from 28.80 to 58.18 years. Comparatively, the median age for the no reinsertion group is slightly lower at 48.18 years, while it is higher for the reinsertion group at 51.69 years (*P* = .205). Of the total patients, 139 are male, and 115 are female. In the no reinsertion group, 54.5% are male, compared to 57.1% in the reinsertion group (*P* = .816). The body mass index for all groups shows a median of 21.0 kg/m^2^, with small variations in the interquartile range between the no reinsertion (18.0–23.0) and reinsertion groups (19.0–23.5) (*P* = .538). Most patients underwent an open surgical approach, including thoracotomy (74.0%). Other approaches such as laparoscopic and mini-incision were less common, such as laparoscopic surgery (20.9%), and mini-incision (5.1%). Smoking and drinking habits are fairly similar between groups, with no significant differences noted in the proportions of current smokers and drinkers (*P* = .393 and *P* = .866, respectively). Different diagnoses are represented among the patients, with rheumatic valvular heart disease being the most common at 48.8%. The proportion of patients with coronary artery disease complicated with heart valve disease is notably higher in the reinsertion group (23.8%) compared to the no reinsertion group (11.2%). No significant differences were observed between the non-reinsertion and reinsertion groups in terms of age, gender, body mass index, surgical approach, smoking and drinking habits and diagnosis.

**Table 1:** Demographic and clinical characteristics of the cardiac surgical patients.

Characteristics	All patients	No reinsertion (n = 233)	Reinsertion (n = 21)	*Z/χ* ^*2*^	*P*
Age, yr, median (IQR)	48.78 (28.80–58.18)	48.18 (24.06–58.15)	51.69 (37.93–60.61)	−1.267	.205
Gender, *n* (%)					
Male	139.0	127.0 (54.5)	12.0 (57.1)	0.054	.816
Female	115.0	106.0 (45.5)	9.0 (42.9)		
BMI, kg/m^2^, median (IQR)	21.0 (18.0–23.0)	21.0 (18.0–23.0)	21.0 (19.0–23.5)	−0.616	.538
Diagnosis, *n* (%)					
Coronary heart disease	11.0 (4.3)	11.0 (4.7)	0	4.833	.437
Rheumatic valvular heart disease	124.0 (48.8)	113.0 (48.5)	11.0 (52.4)		
Coronary artery disease complicated with heart valve disease	31.0 (12.2)	26.0 (11.2)	5.0 (23.8)		
Congenital heart disease	78.0 (30.7)	73.0 (31.3)	5.0 (23.8)		
Aortic dissection	8.0 (3.1)	8.0 (3.4)	0		
Cardiac tumor	2.0 (0.8)	2.0 (0.9)			
Current smokers, *n* (%)	63.0 (24.8)	58.0 (24.9)	5.0 (23.8)	0.731	.393
Current drinking, *n* (%)	52.0 (20.5)	48.0 (20.6)	4.0 (19.0)	0.029	.866
Diabetes, *n* (%)	11.0 (4.3)	7.0 (3.0)	4.0 (19.0)	11.967	.001
Hypertension, *n* (%)	54.0 (21.3)	48.0 (20.6)	6.0 (28.6)	0.731	.393
Urinary diseases, *n* (%)	26.0 (10.2)	24.0 (10.3)	2.0 (9.5)	0.013	.910
Surgical approach, *n* (%)					
Open	188.0 (74.0)	173.0 (74.2)	15.0 (71.4)	1.844	.398
Laparoscopic	53.0 (20.9)	47.0 (20.2)	6.0 (28.6)		
Mini-incision	13.0 (5.1)	13.0 (5.6)	0		

Statistical significance is indicated by *P*-values, with values <.05 considered significant.

BMI = body mass index, IQR = interquartile range.

Notably, Diabetes shows a significant difference, with 19% in the reinsertion group compared to only 3% in the no reinsertion group (*P* = .001). This significant *P*-value suggests a strong association between diabetes and the likelihood of reinsertion. Diabetic patients also exhibited longer operative times (6.1 hours vs 4.9 hours, *P* = .007) and were generally older (57 vs 47 years, *P* = .006). Significant differences were observed between diabetic and nondiabetic patients in preoperative (66.8 mL/min vs 76.8 mL/min, *P* = .048) and postoperative CCr (50.35 mL/min vs 70.8 mL/min, *P* = .012). Hypertension and urinary diseases show no significant differences.

Table 2 offers an extensive comparison of clinical and operational characteristics between cardiac surgery patients who required reinsertion (n = 21) and those who did not (n = 233). Patients who did not require reinsertion had a median urinary catheter duration of 60.65 hours, substantially higher than the 35.03 hours for the reinsertion group. This difference is statistically significant (*P* = .034), suggesting a shorter duration of urinary catheter use in patients requiring reinsertion. The median duration on ventilators was 19 hours for the no reinsertion group and slightly higher at 21.59 hours for the reinsertion group (*P* = .802). The duration of cardiopulmonary bypass was slightly lower for the reinsertion group (102.5 minutes) compared to the no reinsertion group (115 minutes), but this difference was not significant (*P* = .778). Operative Time: Both groups had similar operative times, around 4.9 hours for the no reinsertion group and 5.08 hours for the reinsertion group, with no significant difference (*P* = .805). The median ICU stay was longer for the no reinsertion group (37.23 hours) compared to the reinsertion group (23.5 hours) (*P* = .138). Days spent in the hospital were similar between the groups (*P* = .408). No significant differences were found between the groups in terms of cardiopulmonary bypass time, operative time, length of ICU stay, and total hospital days. Both preoperative and postoperative creatinine and CCr measurements showed no significant differences between the groups. Measures of daily living activities and pain showed no significant differences between the groups.

**Table 2:** Comparison of characteristics between the reinsertion and no reinsertion groups in cardiac surgery patients.

Characteristics	No reinsertion (n = 233)	Reinsertion (n = 21)	*Z/χ* ^*2*^	*P*
Urinary catheter duration, h	60.65 (36.45–97.51)	35.03 (24.04–68.21)	−2.115	.034
Ventilator duration, h	19.0 (12.70–35.82)	21.59 (10.5–37.5)	−0.251	.802
Cardiopulmonary bypass time, min	115 (87.75–159.0)	102.5 (81.0–176.75)	−0.282	.778
POD catheter removal, n (%)				
POD 0–1	54.0 (23.2)	11.0 (52.4)	8.629	.003
POD >1	179.0 (76.8)	10.0 (47.6)		
Operative time, h	4.9 (4.18–6.0)	5.08 (4.12–6.03)	−0.247	.805
Length of ICU stay, h	37.23 (19.58–75.62)	23.5 (16.22–60.88)	−1.484	.138
Hospital days, d	14.0 (11.0–17.0)	15.0 (11.5–18.0)	−0.827	.408
Drainage tube, number	1.0 (0–2.0)	2.0 (1.0–2.0)	−1.786	.074
Preoperative CCr	76.8 (64.9–88.0)	72.9 (66.2–85.0)	−0.730	.465
Preoperative creatinine, µmol/L	68.5 (52.25–86.0)	68.0 (61.75–84.75)	−0.954	.340
Postoperative CCr	68.4 (51.55–94.05)	54.4 (40.5–86.4)	−1.004	.315
Postoperative creatinine, µmol/L	75.3 (51.25–106.75)	79.0 (63.0–127.5)	−0.954	.340
ADL	50.0 (45.0–55.0)	47.5 (41.25–58.75)	−0.230	.818
NRS	1.0 (1.0–2.0)	1.0 (1.0–2.0)	−0.118	.906

Statistical significance is indicated by *P*-values, with values <.05 considered significant.

ADL = activities of daily living, CCr = creatinine clearance rate, ICU = intensive care unit, NRS = numeric rating scale, POD = postoperative days.

Patients in the reinsertion group generally had more drainage tubes (median of 2) compared to those in the no reinsertion group (median of 1), with this approaching significance (*P* = .074). Patients were categorized based on urinary catheterization duration into early removal (POD (postoperative day) < 1) and late removal (POD > 1) groups. A significantly higher percentage of the reinsertion group (52.4%) had their catheters removed within the first postoperative day compared to 23.2% in the no reinsertion group. This difference is statistically significant (*P* = .003). A majority of the no reinsertion group had their catheters removed after the first postoperative day, contrasting with less than half in the reinsertion group.

Table 3 provides a logistic regression analysis that investigates the risk factors contributing to the need for urinary catheter reinsertion after cardiac surgery. The analysis shows that each additional year increases the risk of reinsertion by 3.6%, with an OR of 1.036 (95% CI = 1.005–1.068) and a statistically significant *P*-value of .023. Patients who have their catheters removed on the first postoperative day are approximately 8.6 times more likely to need reinsertion, as indicated by an OR of 8.570 (95% CI = 2.801–26.223) and a highly significant *P*-value of .001. Diabetic patients face substantially higher risks, with an OR of 8.755 (95% CI = 1.973–38.851), suggesting an approximately 8.8 times increased likelihood of needing catheter reinsertion, which is significant at *P* = .004. The model’s constant of −5.018, with an associated OR of 0.007 and a *P*-value of .001, indicates a low baseline probability of reinsertion when all predictors are at their reference levels.

**Table 3:** Logistic regression analysis for risk factors associated with urinary catheter reinsertion.

Independent variables	Beta Coeff.	SE	Wald	Odds ratio (95% CI)	*P*
Age	0.035	0.015	5.20	1.036 (1.005–1.068)	.023
POD < 1	2.148	0.571	14.173	8.570 (2.801–26.223)	.001
Diabetes	2.17	0.760	8.144	8.755 (1.973–38.851)	.004
Constant	−5.018	0.927	29.334	0.007	.001

Statistical significance is indicated by *P*-values, with values <.05 considered significant.

POD = postoperative days.

## 4. Discussion

Our study revealed an 8.26% rate of urinary catheter reinsertion post-cardiac surgery, aligning with previous findings.^[13]^ However, this rate contrasts with the 21.8% observed in thoracic surgery patients^[6]^ and varies significantly from rates reported in joint arthroplasty (10.7%–84%),^[14]^ anorectal surgery (1%–52%), hernia repair (5.9%–38%),^[15]^ and gynecological surgery.^[16]^ These variations could be attributed to differences in surgical types, operative durations, and postoperative pain intensities. Notably, anorectal and gynecological surgeries, which disrupt bladder innervation, pose higher risks for POUR.

A significant observation in our study was that over half of the patients with urinary retention were above 50 years of age. Multivariable logistic regression analysis identified increased age as a risk factor for catheter reinsertion. This is corroborated by previous studies, where being over 50 was a significant risk factor for POUR (OR of 1.97),^[17]^ and patients over 60 faced a higher risk (OR of 7.09).^[18]^ In our study, each additional year of age increased the risk of POUR by approximately 3.6% (OR of 1.036), suggesting that age-related neuronal degeneration may contribute to bladder dysfunction in older patients.^[7]^

Diabetes emerged as another significant risk factor for catheter reinsertion. Our findings showed that diabetic patients were 8.755 times more likely to require reinsertion than nondiabetic patients, echoing previous research.^[19,20]^ Studies have identified diabetes as an independent predictor of urinary retention due to impaired bladder sensation and contractility.^[3,21]^ Our study also noted that diabetic patients were generally older with longer operative times (6.1 hours vs 4.9 hours) and lower CCr (50.35 mL/min vs 70.8 mL/min), hinting at a potential link between diabetes, renal function, and POUR.

Early removal of the urinary catheter was independently associated with a higher risk of reinsertion, consistent with existing literature.^[4,6,22–24]^ Our data showed that 52.4% of patients who had their catheters removed on the first postoperative day required reinsertion. While the length of hospital stay was longer in the reinsertion group, this did not reach statistical significance. These findings underscore the need for careful consideration regarding the timing of catheter removal, especially in older patients and those with diabetes.

Interestingly, the presence of drainage tubes post-surgery did not increase the risk of catheter reinsertion. Despite causing discomfort and restricting mobility, they did not contribute to POUR. Moreover, pain levels reported post-surgery were mild and did not correlate with POUR, aligning with prior research.^[1]^

## 5. Conclusion

Our study highlights the complexity of managing urinary catheterization post-cardiac surgery. Factors such as age, diabetes, and the early timing of catheter removal play crucial roles in patient outcomes. Notably, our findings underscore that older patients and those with diabetes face significantly higher risks, with diabetics being approximately 8.755 times more likely to require reinsertion. Additionally, removing the catheter on the first postoperative day significantly increases the likelihood of needing reinsertion. These insights can inform clinical decision-making, emphasizing the need for individualized patient care to minimize the risk of POUR. Further investigation is warranted to better understand the specific impacts of operative times and renal function on the risk of POUR in these high-risk groups. Future research should continue to explore the interplay between these factors to optimize postoperative care in cardiac surgery patients.

## 6. Limitations

Our study acknowledges several limitations. Firstly, its execution in a single hospital setting may restrict the generalizability of our results. To enhance the applicability of our findings, future research should be conducted across multiple centers, thereby providing more comprehensive evidence. Secondly, our study did not account for the potential effects of certain medications on urinary retention, particularly drugs with anticholinergic properties, opioids, and nonsteroidal anti-inflammatories (NSAIDs).^[1,25]^ Anticholinergics, known to inhibit detrusor contractility,^[1]^ and opioids, which decrease bladder contractility and sensation,^[26]^ along with NSAIDs that reduce bladder contractility through cyclooxygenase-2 inhibition,^[27]^ warrant further investigation to understand their impact on urinary re-catheterization in cardiac patients. Thirdly, the optimal timing for urinary catheter removal continues to be debated. Our study did not conclusively determine the most appropriate timing for catheter removal, indicating a need for additional research to establish evidence-based guidelines to aid in clinical decision-making.

## Acknowledgments

We extend our sincere gratitude to all participants who took part in this study.

## Author contributions

**Formal analysis:** Si-Si Deng, Sheng-Sheng Zhong.

**Funding acquisition:** Si-Si Deng, Xin-Huan Lu.

**Methodology:** Si-Si Deng, Sheng-Sheng Zhong.

**Resources:** Xin-Huan Lu.

**Writing – original draft:** Si-Si Deng, Sheng-Sheng Zhong.

**Writing – review & editing:** Si-Si Deng, Xin-Huan Lu.

## References

[1] Kowalik U Plante MK. Urinary retention in surgical patients. Surg Clin North Am. 2016;96:453–67. 27261788 10.1016/j.suc.2016.02.004

[2] Altschul D Kobets A Nakhla J. Postoperative urinary retention in patients undergoing elective spinal surgery. J Neurosurg. 2017;26:229–34. 10.3171/2016.8.SPINE151371 27767680

[3] Sung KH Lee KM Chung CY. What are the risk factors associated with urinary retention after orthopaedic surgery? Biomed Res Int. 2015;2015:613216. 25789322 10.1155/2015/613216 PMC4348600

[4] Hiraki M Tanaka T Sadashima E Sato H Kitahara K. The risk factors of acute urinary retention after laparoscopic colorectal cancer surgery in elderly patients receiving epidural analgesia. Int J Colorectal Dis. 2021;36:1853–9. 33907859 10.1007/s00384-021-03938-2

[5] Griesdale DE Neufeld J Dhillon D. Risk factors for urinary retention after hip or knee replacement: a cohort study. Can J Anaesth. 2011;58:1097–104. 21989549 10.1007/s12630-011-9595-2 PMC3219865

[6] Young J Geraci T Milman S Maslow A Jones RN Ng T. Risk factors for reinsertion of urinary catheter after early removal in thoracic surgical patients. J Thorac Cardiovasc Surg. 2018;156:430–5. 29609886 10.1016/j.jtcvs.2018.02.076

[7] Baldini G Bagry H Aprikian A Carli F Warner DS Warner MA. Postoperative urinary retention: anesthetic and perioperative considerations. Anesthesiology. 2009;110:1139–57. 19352147 10.1097/ALN.0b013e31819f7aea

[8] Chang Y Chi K-Y Tai T-W. Risk factors for postoperative urinary retention following elective spine surgery: a meta-analysis. Spine J. 2021;21:1802–11. 34015508 10.1016/j.spinee.2021.05.009

[9] Saint S Greene MT Krein SL. A program to prevent catheter-associated urinary tract infection in acute care. N Engl J Med. 2016;374:2111–9. 27248619 10.1056/NEJMoa1504906 PMC9661888

[10] Sun Z Xu Y. Medical Statistics. Beijing: People’s Medical Publishing House; 2014.

[11] Barthel D. Functional evaluation: the Barthel index, Maryland State. Med J. 1965;14:16–65. 14258950

[12] Deschka H Machner M Welp H Dell’Aquila AM Erler S Wimmer‐Greinecker G. Cardiac reoperations in octogenarians: do they really benefit? Geriatr Gerontol Int. 2016;16:1138–44. 26460153 10.1111/ggi.12609

[13] Eriksen JR Munk-Madsen P Kehlet H Gögenur I. Postoperative urinary retention after laparoscopic colorectal resection with early catheter removal: a prospective observational study. World J Surg. 2019;43:2090–8. 30993391 10.1007/s00268-019-05010-1

[14] Markopoulos G Kitridis D Tsikopoulos K Georgiannos D Bisbinas I. Bladder training prior to urinary catheter removal in total joint arthroplasty. A randomized controlled trial. Int J Nurs Stud. 2019;89:14–7. 30316955 10.1016/j.ijnurstu.2018.09.007

[15] Jackson J Davies P Leggett N. Systematic review of interventions for the prevention and treatment of postoperative urinary retention. BJS Open. 2019;3:11–23. 30734011 10.1002/bjs5.50114 PMC6354194

[16] Kandadai P Saini J Patterson D O’Dell K Flynn M. Urinary retention after hysterectomy and postoperative analgesic use. Female Pelvic Med Reconstr Surg. 2015;21:257–62. 25521470 10.1097/SPV.0000000000000151

[17] Hansen B Søreide E Warland A Nilsen O. Risk factors of post-operative urinary retention in hospitalised patients. Acta Anaesthesiol Scand. 2011;55:545–8. 21418152 10.1111/j.1399-6576.2011.02416.x

[18] Mason SE Scott AJ Mayer E Purkayastha S. Patient-related risk factors for urinary retention following ambulatory general surgery: a systematic review and meta-analysis. Am J Surg. 2016;211:1126–34. 26257154 10.1016/j.amjsurg.2015.04.021

[19] Dreijer B Møller MH Bartholdy J. Post-operative urinary retention in a general surgical population. Eur J Anaesthesiol. 2011;28:190–4. 21206278 10.1097/EJA.0b013e328341ac3b

[20] Roadman D Helm M Goldblatt MI Kindel TL Gould JC Higgins RM. Postoperative urinary retention after bariatric surgery: an institutional analysis. J Surg Res. 2019;243:83–9. 31170554 10.1016/j.jss.2019.05.005

[21] Boitano LT DeBono M Tanious A. Incidence of and risk factors for postoperative urinary retention in men after carotid endarterectomy. J Vasc Surg. 2020;72:943–50. 31964571 10.1016/j.jvs.2019.10.093

[22] Tam V Lutfi W Morgan K. Impact of enhanced recovery pathways and early urinary catheter removal on post-operative urinary retention. Am J Surg. 2020;220:1264–9. 32680619 10.1016/j.amjsurg.2020.06.057

[23] Benoist S Panis Y Denet C Mauvais F Mariani P Valleur P. Optimal duration of urinary drainage after rectal resection: a randomized controlled trial. Surgery. 1999;125:135–41. 10026745

[24] Kwaan MR Lee JT Rothenberger DA Melton GB Madoff RD. Early removal of urinary catheters after rectal surgery is associated with increased urinary retention. Dis Colon Rectum. 2015;58:401–5. 25751796 10.1097/DCR.0000000000000317

[25] Verhamme KM Sturkenboom MC Stricker BHC Bosch R. Drug-induced urinary retention: incidence, management and prevention. Drug Saf. 2008;31:373–88. 18422378 10.2165/00002018-200831050-00002

[26] Kuipers PW Kamphuis ET van Venrooij GE. Intrathecal opioids and lower urinary tract function: a urodynamic evaluation. Anesthesiology. 2004;100:1497–503. 15166570 10.1097/00000542-200406000-00023

[27] Verhamme KM Dieleman JP Van Wijk MA. Nonsteroidal anti-inflammatory drugs and increased risk of acute urinary retention. Arch Intern Med. 2005;165:1547–51. 16009872 10.1001/archinte.165.13.1547

